# Conformational Dynamics of Actin: Effectors and Implications for Biological Function

**DOI:** 10.1002/cm.20473

**Published:** 2010-07-29

**Authors:** Gábor Hild, Beáta Bugyi, Miklós Nyitrai

**Affiliations:** Department of Biophysics, University of Pécs, Faculty of MedicinePécs, Szigeti str. 12, H-7624, Hungary

**Keywords:** protein structure, cytoskeleton, actin, actin-binding proteins, mutations, diseases

## Abstract

Actin is a protein abundant in many cell types. Decades of investigations have provided evidence that it has many functions in living cells. The diverse morphology and dynamics of actin structures adapted to versatile cellular functions is established by a large repertoire of actin-binding proteins. The proper interactions with these proteins assume effective molecular adaptations from actin, in which its conformational transitions play essential role. This review attempts to summarise our current knowledge regarding the coupling between the conformational states of actin and its biological function.

## Introduction

Proteins are essential building blocks of the living systems. They are involved in all the biological functions playing structural and/or regulatory roles. In most of the cases proteins are required to be adaptive, i.e., to change their structure and function under certain cellular conditions. A frequent example of this adaptation is the up and down regulation of their activity, which occurs in many cases through interactions with other proteins and/or small regulatory molecules. The conformational elasticity of the proteins is essential for their adaptive properties. In many cases the details of the relationship between the various conformational states and the functions of proteins are well established. In some cases the characterisation of the coupling between the structural transitions and the modifications in the biological function is not complete. As proteins are often central components of cellular machineries, the detailed description of their conformational dynamics is desirable.

The interest in actin has extensively increased since its discovery by Straub [ 1942]. In their pioneering work Straub et al. extracted actin from muscle tissue, where actin is the major constituent of the thin filaments and participates in the muscle contraction as a working partner of the thick filament forming myosin [Bagshaw, 1992; Geeves and Holmes, 2005]. Apart from its function in muscle, actin is also proved to be an essential constituent of the cytoskeleton of various different cell types [Sheterline et al., 1995] besides microtubules and intermediate filaments [Wilson, 1982; Howard and Hyman, 2003; Oshima, 2007]. The actin cytoskeleton or the microfilament system has a remarkably versatile role in supporting diverse processes such as establishing and maintaining cellular polarity, driving cell shape changes, cell motility, adhesion, cytokinesis, endocytosis and intracellular trafficking [Pollard and Cooper, 2009].

After some debate since the first report on actin as an intranuclear entity dated back to the 60s [Lane, 1969] actin also became generally accepted as an important structural and functional component of the cell nucleus [Rando et al., 2000; Pederson and Aebi, 2002; Pederson and Aebi, 2005; McDonald et al., 2006; Schleicher and Jockusch, 2008]. In the nucleus actin is involved in transcription [Pederson and Aebi, 2005; Miralles and Visa, 2006; Percipalle and Visa, 2006], in chromatin remodelling [Rando et al., 2000] and also signal transduction [Vartiainen et al., 2007].

Actin is not restricted to metazoans but can also be found in plants. Since the discovery of the actomyosin complex in plants [Vorobeva and Poglazov, 1963] the function of plant actin has been demonstrated in intracellular movement of organelles and vesicles [Takagi, 2003], exo- and endocytosis and in the plant's cell cycle [Wick, 1991; Kost et al., 2002]. The recent discovery of the bacterial actin homologues, ParM and MreB [Jones et al., 2001; van den Ent et al., 2001], established the presence of the prokaryotic actin cytoskeleton, which mediates processes such as plasmid segregation [Jensen and Gerdes, 1999; van den Ent et al., 2002] and cell shape regulation [Doi et al., 1988; Jones et al., 2001; Lee and Stewart, 2003].

Actin has a large repertoire of interacting partners including metal ions, nucleotides and actin-binding proteins, which attribute versatile functions to actin [Sheterline et al., 1995; Lappalainen, 2007]. Several studies provided convincing evidence that both monomeric and filamentous actin could have different conformations depending on the bound molecule and these conformational changes were implicated in the functions of actin.

It is essential to understand how actin can adapt and modify its conformation for the various biological processes, what cellular components are effective to regulate these conformational transitions and how these different structural states are coupled to the complex and tightly regulated mechanisms through which actin fulfils its manifold biological activity. Here we review the present knowledge on the conformational dynamics of actin from structural, spectroscopic and cellular biology studies of complexes that actin forms with its interacting partners. We will also attempt to discuss how these conformational differences contribute to the functional segregation of actin structures in living cells.

## Structure of Actin

Since its discovery in 1942 [Straub, 1942] actin has seen a long and fruitful period of investigations. The simple and rapid purification of actin [Spudich and Watt, 1971] from acetone powder of muscle tissues was established many years ago [Feuer et al., 1948]. These methods, which make it possible to obtain actin in high quantities, boosted the exploration of the biological functions of muscle actin. Protocols for the preparation of actin from nonmuscle sources is also available [Schafer et al., 1998; Joel et al., 2004]. Most of the results reviewed here are related to the best characterised muscle actin, however, the existing data related to nonmuscle actin isoforms will also be discussed.

**Fig. 1:**
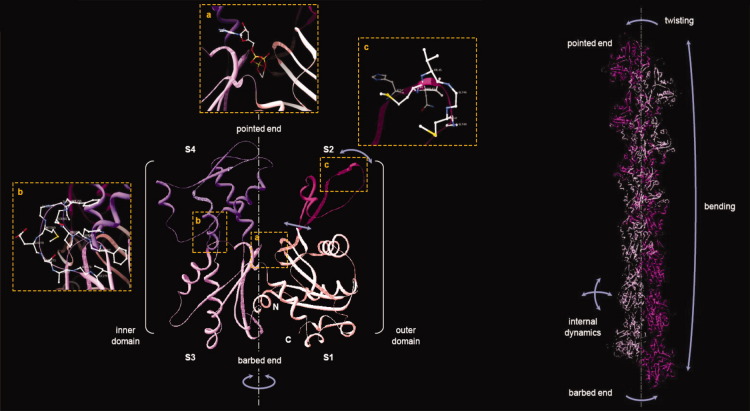
Overview of the structure and conformational dynamics of actin monomer and filament **(A)** Crystal structure of the G-actin molecule. Actin subdomains are indicated by different colors and numbers according to Holmes et al (S1) amino acids 1–32, 70–144, 338–375; (S2) amino acids 33–69; (S3) amino acids 145–180, 270–337; (S4) amino acids 181–269 [Holmes et al., 1990]. The amino-, and carboxyl termini of the molecule are indicated by N and C, respectively. Orange-dotted boxes depict enlarged views of the nucleotide binding cleft with bound ATP (a), the structure of the hydrophobic loop (b, amino acids 262–274, rotated by 90° to the right with respect to the main axis of the monomer, shown by gray dashed-dotted line) and the DNase binding loop (c, amino acids 40–48). The schematic representation of the conformational dynamics on the basis of the normal mode analysis of G-actin is shown by blue arrows [Tirion and ben-Avraham, 1993]. The image was made on the basis of the crystal structure of rabbit skeletal muscle actin in complex with DNase I (PDB code 1ATN, [Kabsch et al., 1990]). (**B**) Helical organization of actin protomers in the filament. A structural model of a 13-mer F-actin was derived from Oda's F-actin protomer model (PDB code 2ZWH, [Oda et al., 2009]). The two linear strands of actin protomers composing the two-start right-handed long pitch helix are colored by dim and dark violet, respectively. The single-start left-handed short pith helix is assembled by alternating dim and dark actin protomers. The schematic representation of the conformational dynamics of F-actin is shown by blue arrows. The ribbon diagrams were obtained with Deep View/Swiss PDB Viewer.

Actin exists in both monomeric (globular or G-actin) and polymeric (filamentous or F-actin) form (Fig. 1.). The first atomic-resolution 3D structure of G-actin in complex with DNase I was reported in 1990 by [Kabsch et al., 1990] (PDB:1ATN). The structure revealed that the actin monomer has a cubic-like shape with a size of 6.7 × 4.0 × 3.4 nm. The monomer can be divided into two main domains, referred to as inner and outer domains on the basis of their position relative to the axis of the filament. Each of the main domains is composed of two subdomains (S1–4) (Fig. 1A). The main domains are separated by a cleft containing a tightly bound adenosine-derived nucleotide in complex with a divalent cation, which is thought to be magnesium in the physiological state of actin [Estes et al., 1992](Fig. 1A inset a).

Under physiological ionic conditions actin monomers assemble into filaments. Actin polymerisation proceeds through kinetically distinct steps. It is initiated by the slow formation of actin nuclei (dimers/trimers), which serve as seeds for the subsequent filament elongation. During elongation more actin monomers associate to than dissociate from either of the two ends, which results in the net growth of both filament ends. The steady-state phase is characterised by a dynamic equilibrium where the length of the actin filaments remains constant, while actin monomers continually associate to and dissociate from the ends. In this dynamic equilibrium a stationery population of free actin monomers is established, called critical concentration, whose value lies between the critical concentration of the barbed and pointed end. Besides structural polarity, determined by the arrangement of actin protomers within the filament, actin filaments also exhibit a kinetic polarity, which is defined by the different monomer association and dissociation rates at the two ends. The barbed or plus end binds actin monomers faster than the pointed or minus end. Although its ATPase activity is not crucial for actin polymerisation, actin self-assembly is associated with the ATPase cycle, which powers treadmilling process [Wegner, 1976].

The first high-resolution structural model of the actin filament at a resolution of 8 Å was proposed by [Holmes et al., 1990]. This structure was obtained by fitting the atomic structure of G-actin by simple rigid body rotation into the experimentally obtained X-ray fibre diffraction pattern of oriented F-actin gels. Recently, an improved high-resolution F-actin model was achieved by [Oda et al., 2009] with a resolution of 3.3 Å in the radial and 5.6 Å in the equatorial directions. The repetitive, polarized arrangements of actin protomers within the filaments defines a double-stranded, right-handed helix with a half-pitch of 37 nm and a one-start left-handed genetic helix with a rise of 2.75 nm per monomer (Fig. 1B). The width of the filament is within the range of 7–10 nm. These results gave excellent framework for subsequent investigations by describing the most important structural details and geometrical properties of actin filaments. However, one should keep in mind that these are models based on the average mass distributions observed in structural studies. The physically veritable existing actin filament conformations deviate from these ideal structural models due to the presence of inherent structural disorders built into the filaments [Egelman et al., 1983; Egelman and DeRosier, 1991, 1992].

## Methods to Investigate the Conformational Dynamics of Actin

**Fig. 2:**
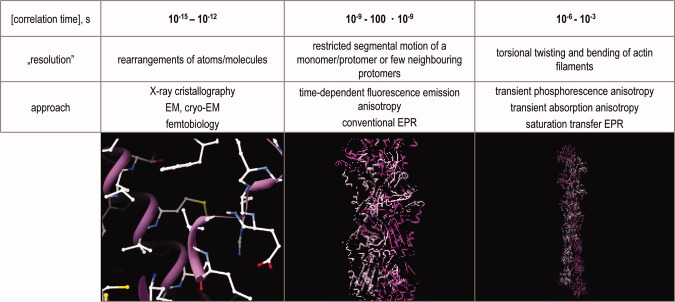
Summary of the conformational changes in actin The table shows the corresponding correlation times and the suitable approaches for their investigation.

**Fig. 3:**
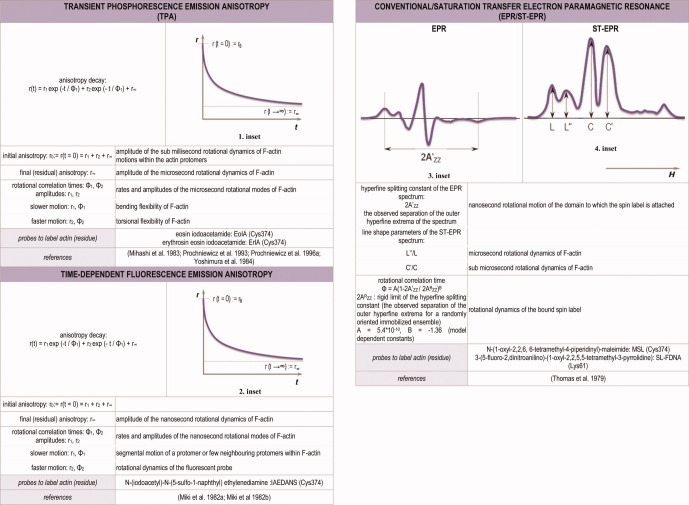
Summary of the most commonly used spectroscopic approaches to study the conformational dynamics of actin The formulation and parameters of transient phosphorescence emission anisotropy (TPA), time-dependent fluorescence emission anisotropy and conventional/saturation transfer (ST) EPR. Typical phosphorescence (1. inset)/fluorescence (2. inset) anisotropy decay (*r*(*t*)) of actin, and typical EPR (3. inset)/ST-EPR (4. inset) spectra of actin are shown. *H* is releated to the direction and the strength of the applied magnetic field. In phosphorescence/fluorescence emission anisotropy the kinetics of anisotropy decay, while in EPR/ST-EPR the shape of the spectrum characteristic for the conformational dynamics of the molecule. [Color figure can be viewed in the online issue which is available at http://wileyonlinelibrary.com.]

Rather than being rigid structures, both monomeric and filamentous actin have remarkable conformational flexibility and adopt many various structural states in response to interaction with their partner molecules [Orlova and Egelman, 1995; Orlova et al., 1995; Schuler, 2001]. The various conformational changes occurring in actin are characterized by different correlation times distributed on a broad time scale (Fig. 2). The suitable experimental methods sensitive to these different modes of motions in actin are well established (Fig. 2). Correlation times on the *fs* range reflect the rearrangements of atoms/molecules, and these structural changes can be resolved by X-ray crystallography, electron microscopy (EM), cryo-EM and femtobiological approaches [Egelman, 2000; Resch et al., 2002; Sundstrom, 2008]. The *ns* correlation times are related to the change in the restricted segmental motion of a monomer/protomer or a few neighbouring protomers and can be determined by time-dependent fluorescence anisotropy [Ikkai et al., 1979; Miki et al., 1982a, b] or conventional electron paramagnetic resonance (EPR) [Thomas et al., 1979; Mossakowska et al., 1988]. The torsional twisting and bending motions of the whole actin filament characterised by correlation times in the μs and μs–ms range, can be described by phosphorescence anisotropy [Prochniewicz et al., 1996a; Yoshimura et al., 1984], saturation transfer (ST) EPR [Thomas et al., 1979; Hegyi et al., 1988], and transient absorption anisotropy measurements [Mihashi et al., 1983]. A specific method—temperature dependent Förster-type resonance energy transfer (FRET)—was described to characterise the flexibility of the proteins [Somogyi et al., 1984; Somogyi et al., 2000]. Due to the nature of the method it is sensitive to all kinds of intramolecular motions, which alter the relative distance or relative fluctuations of the donor and acceptor molecules. The most widely used spectroscopic approaches suitable for investigating the conformational dynamics of actin are summarized in Figure 3. The aromatic amino acids in actin as intrinsic probes, or extrinsic fluorescent chemical compounds, which can be covalently attached to specific residues of actin, can also report the existence of local conformational changes within the protein matrix of monomers/protomers. The spectral properties of the fluorescent probes (emission spectra, quantum yield, lifetime, anisotropy) are sensitive to the changes in its local environment, providing further experimental tools for the analyses of structural changes in actin [Lakowicz, 2006].

## Self-Assembly of Actin and its Interactions with Nucleotides and Cations

The main ligands that bind to the central cleft of the actin monomers are an adenosine nucleotide and a divalent cation (Fig. 1A inset a) [Sheterline et al., 1995]. The single nucleotide-binding site binds ATP with a much tighter affinity (*K*_D_ = 10^−10^ M) than ADP (*K*_D_ = 10^−8^ M) [Neidl and Engel, 1979; Brenner and Korn, 1981]. The single high affinity cation-binding site (*K*_D_ ∼10^−9^ M) is thought to be occupied by magnesium in cells [Estes et al., 1992]. G-actin has a low ATPase activity (0.6 h^−1^) [Schuler, 2001], which is accelerated significantly as ATP-bound actin monomers incorporate into the filaments [Pollard and Weeds 1984; Pantaloni et al., 1985]. In the fast polymerisation regime of muscle actin, the kinetics of actin assembly and ATP hydrolysis is faster than the subsequent release of the γ-phosphate (*P*_*i*_). This results in the appearance of an ATP/ADP-*P*_*i*_ cap at the barbed end, while the rest of the filament contains ADP-bound actin protomers [Brenner and Korn, 1981; Carlier and Pantaloni, 1986; Carlier et al., 1987; Korn et al., 1987]. In contrast, under similar conditions yeast actin polymerises and releases the hydrolysed *P*_*i*_ almost simultaneously, which results in homogeneous ADP-bound actin protomers along the whole filament [Yao et al., 1999; Yao and Rubenstein, 2001].

The Holmes model postulated the importance of an interstrand hydrophobic plug-pocket interaction in filament integrity [Holmes et al., 1990]. In actin monomers a hydrophobic loop of residues 262–274 (for muscle actin, Fig. 1A inset b) between S3 and S4 lies tightly in a parked position near the main body of S4. Holmes et al. proposed that upon G-to-F transition this loop underwent a conformational change forming a hydrophobic plug (266–269). This plug extends perpendicular to the filament axis, and is locked into a hydrophobic pocket formed by two adjacent actin protomers of the opposite strand. Thereby the plug-pocket interaction would stabilise the structure of the actin filaments.

The importance of this cross-strand hydrophobic interaction and loop mobility in actin filament integrity was supported by disulfide cross-linking studies. These experiments showed that mutant G-actin—in which the loop is locked to the protein backbone—could not polymerise [Shvetsov et al., 2002], and cross-linking the loop after filament formation destabilised F-actin [Orlova et al., 2004]. Fluorescence probing of the loop further supported this hypothesis [Feng et al., 1997; Musib et al., 2002]. Mutagenesis studies revealed that decreasing the hydrophobicity of the loop resulted in cold sensitive polymerisation incompetent actin mutants, which demonstrates that the loop hydrophobicity is important for filament formation [Chen et al., 1993; Kuang and Rubenstein, 1997]. However, contradicting with the plug-pocket hypothesis, disturbing the hydrophobicity of the plug by replacing amino acids with negatively charged residues caused more pronounced effects at its C-terminus than at the N-terminus [Kuang and Rubenstein, 1997]. The N-terminus is assumed to be associated more extensively to the pocket via hydropobic interactions in Holmes's F-actin model. On the basis of these results the authors suggested that the hydrophobic plug can exhibit dynamic fluctuations and adopt different conformational states rather than being tightly locked in the pocket [Tirion et al., 1995]. Further analysis of the dynamics of the loop with cross-linking and EPR spectroscopy revealed that the loop was indeed highly mobile in both G- and F-actin [Shvetsov et al., 2006]. The interspin distance was similar in both G- and F-actin, but the distribution was broader in F-actin compared to G-actin, which suggests that the loop occupies predominantly a parked, less extended position than proposed by the Holmes model (even in F-actin) and its dynamics correlates with G-to-F transition [Scoville et al., 2006; Shvetsov et al., 2006]. The less extended position of the loop in F-actin is in agreement with the improved F-actin model that predicts a smaller filament diameter (with a radius of gyration of 23.7 Å) compared to Holmes-model's radius of gyration of 25 Å [Oda et al., 2009]. This narrower interstrand gap does not require the hydrophobic plug to largely change its conformation upon G-to-F transition.

The influence of the different nucleotides on the conformational dynamics of actin filaments was first demonstrated by the analysis of EM images of negatively stained actin filaments, and by dynamic elasticity and viscosity measurements of solutions of F-actin. These measurements suggested that actin filaments formed from ATP-actin monomers were more rigid than actin filaments assembled from ADP-actin monomers [Janmey et al., 1990]. Based on these results it was proposed that the energy released upon ATP hydrolysis is coupled to conformational dynamics changes and stabilization of the structure of F-actin [Janmey et al., 1990]. Although this finding was debated later [Pollard et al., 1992; Newman et al., 1993], several subsequent analysis further supported the nucleotide-dependent conformational changes of actin. Steady-state phosphorescence measurements showed that actin filaments polymerised from Mg^2+^-ADP actin monomers had lower steady-state phosphorescence emission anisotropy, and thus greater torsional flexibility than filaments polymerised from Mg^2+^-ATP actin monomers [Rebello and Ludescher, 1998]. Temperature dependent FRET measurements revealed nucleotide-induced local conformational changes around *Cys*^*374*^ in S1 and substantially greater intermonomer flexibility of filaments that were assembled from ADP-actin monomers than those assembled from ATP-actin monomers. These data suggest that more tenuous interprotomer connections are formed when the filament is polymerized from ADP-bound monomers [Nyitrai et al., 2000]. Consistently, differential scanning calorimetry (DSC) measurements further supported the more stable structure of actin filaments assembled from ATP-bound actin monomers through their higher thermal stability [Orban et al., 2006].

A detailed light microscopy analysis of the thermal fluctuations of fluorescently labelled F-actin showed that there was no difference in the persistence length of actin filaments assembled from either ATP-actin or ADP-actin monomers (*L*_p_ = 9 μm), which suggests similar bending flexibilities [Isambert et al., 1995]. Addition of BeF_3_—which mimics the γ-phosphate [Combeau and Carlier, 1988]—to F-ADP-actin to reconstitute the F-ADP-*P*_*i*_-state resulted in higher flexural rigidity of F-ADP-*P*_*i*_-actin (*L*_p_ = 13.5 μm) compared to F-ATP, or -ADP-actin [Isambert et al., 1995]. While these observations established the existence of conformational changes related to the assembly and ATPase activity of actin, the exact nature and the sequence of these structural rearrangements have not been revealed so far.

The ATPase activity is thought to be coupled to domain movements in actin [Belmont et al., 1999]. Comparison of the crystal structures of β-actin monomer with an without bound profilin [Schutt et al., 1993; Chik et al., 1996] showed that the nucleotide-binding cleft of G-actin can adopt an ‘open’ or a ‘closed’ conformation. During the close-to-open transition the interdomain cleft opens up by 10°. After the closed and open structures of monomeric actin were docked into EM reconstructions of actin filaments [Belmont et al., 1999], similarities with other NTPases (for review see [Geeves and Holmes, 1999; Sablin and Fletterick, 2001] and with the nucleotide-free structure of the Arp3 (Actin-related protein) subunit of Arp2/3 complex [Robinson et al., 2001] suggested that the close-to-open structural transition was related to nucleotide hydrolysis.

Consistently, FRET measurements showed that the replacement of ATP by ADP in the nucleotide-binding cleft resulted in conformational changes that bring *Gln*^*41*^ and *Cys*^*374*^ closer to each other [Gaszner et al., 1999]. Fluorescence quenching experiments also revealed intraprotomer changes occurring in S1 upon G-to-F-actin transition, which altered the charge distribution around at least one of the four tryptophans (*Trp79*, *Trp86*, *Trp340*, *Trp356* in S1) [Hild et al., 1996]. In contrast, more recent crystal structures of uncomplexed G-actin (mutated and chemically modified actin monomers) directly showed that the nucleotide-binding cleft is closed in both the ADP- [Otterbein et al., 2001] and the ATP-state (mimicked by using AMPPNP) [Graceffa and Dominguez, 2003; Rould et al., 2006]. In support to this, the recently refined model of F-actin shows a closed nucleotide-binding cleft in the filament, and reveals a flattening of the actin molecule upon incorporation into filaments caused by a 20° rotation of the two major domains [Oda et al., 2009]. These structural rearrangements bring *Gln*^*137*^ close to the γ-phosphate of the bound ATP. As *Gln*^*137*^ is implicated in the ATPase mechanism these structural rearrangements may be related to the regulation of ATPase activity of actin [Oda et al., 2009]. To achieve a high resolution F-actin structure Oda et al. used gelsolin to cap actin filaments [Oda et al., 2009], which was shown to induce long range allosteric conformational changes in F-actin [Orlova and Egelman, 1995; Prochniewicz et al., 1996b; Khaitlina and Hinssen, 1997)].

Actin self-assembly and ATPase activity were also shown to alter the conformation of the DNase I binding loop (or D-loop) (Fig. 1A inset c) in S2 by inducing a transition from a flexible loop to an α-helical structure [Otterbein et al., 2001; Graceffa and Dominguez, 2003]. This observation led to the proposal that the helical conformation represent the ADP-state of actin protomers, but its validity has been questioned recently [Rould et al., 2006; Oda et al., 2009]. Comparative structural studies between BeF_3_-F-actin and ADP-F-actin showed that the structure of S2 is more disordered in the ADP-state which results in breaking one of the longitudinal bonds and destabilization of the filament [Orlova and Egelman, 1993].

Tightly bound cations can modify the conformational state of actin as well. Both the torsional and bending flexibilities of Mg^2+^-F-actin were shown to be higher compared to Ca^2+^-actin filaments using spectroscopic and EM approaches [Orlova and Egelman, 1993; Rebello and Ludescher, 1998]. While measuring the flexibility of single actin filaments using optical tweezers showed no significant cation dependence in the flexural rigidity of actin filaments [Tsuda et al., 1996; Yasuda et al., 1996]. In fluorescence spectroscopy measurements the flexibility of the actin filaments [Hild et al., 1998] and also the intra- and interprotomer flexibilities were larger in the case of Ca^2+^-F-actin than for Mg^2+^-F-actin [Nyitrai et al., 1999]. The apparent conflict between these results can be explained by considering that different approaches were used to investigate the dynamic properties of actin filaments and these methods were sensitive to different conformational transitions and modes of motions in the actin filaments.

Temperature-dependent FRET measurements revealed no difference between the flexibility of the outer domain (S1 and S2) of Ca^2+^-G-actin and Mg^2+^-G-actin in the range of 6–26 °C, while above 26 °C a conformational transition was detected in Ca^2+^-actin monomers [Nyitrai et al., 1998]. It was also proved by fluorescence spectroscopy and EPR measurements that the C-terminal part of the monomer became more rigid when the bound calcium is replaced by magnesium [Nyitrai et al., 1997]. The effects of cations are highly influenced by the environmental pH [Hild et al., 2002] that can intracellularly change under physiological (e.g., fatigue) and pathological (e.g., ischemia) conditions [Mohabir et al., 1991; Thompson et al., 1992]. The interprotomer flexibility of Mg^2+^-F-actin was found to be lower than that of Ca^2+^-F-actin in the range between pH 6.5 and pH 7.4 [Hild et al., 2002], while there was no such pH induced difference in the case of Ca^2+^-F-actin. The interprotomer connections were more rigid at both pH 6.5 and 7.4 in Mg^2+^-F-actin than in Ca^2+^-F-actin [Hild et al., 2002].

The persistence length of Mg^2+^-actin filaments was proved to be unaffected (*L*_*p*_ ∼8 μm) when the filaments were polymerized at different pH values (between pH 5 and 9) [Arii and Hatori, 2008]. In contrast, the persistence length of Ca^2+^-F-actin increased from ∼4.5 μm to ∼9 μm as the pH increased from 5 to 9, suggesting higher mobility of actin protomers within Ca^2+^-F-actin at lower pH values [Arii and Hatori, 2008]. The sliding velocity of actin filaments on heavy meromyosin were slower for both Ca^2+^-actin and Mg^2+^-actin filaments as the pH decreased [Kron and Spudich, 1986; Arii and Hatori, 2008]. In experiments with myofibrils it was shown that under isometric conditions the ATPase activity of the myofibrils did not depend on the pH in the range of 6.5–8.0, while the isometric tension has shifted to a slightly higher value as the pH increased [Potma et al., 1994]. This observation and correlation between the sliding velocity and the persistence length of actin filaments suggest that the proper functioning of the sliding machinery is tightly related to the flexibility of its components during muscle contraction [Arii and Hatori, 2008].

The polymerisation of actin filaments was accelerated and the helical pitch between the protomers was increased by lowering the pH [Zimmerle and Frieden, 1988; Wang et al., 1989; Oda et al., 2001]. The pH can also change the properties of the actin monomers. It was found that the binding of calcium to the high affinity binding site became tighter when the pH is decreased from 8 to 6 [Zimmerle and Frieden, 1988].

The observations described above demonstrate that small ligands—such as nucleotides and cations—and also external conditions—such as the pH—modify the conformation of actin. Despite the large amount of accumulated data the biological function of these structural modifications remained somewhat ambiguous. Some observations indicated that ATP hydrolysis altered the thermodynamic and mechanical properties of actin filaments and their interactions with actin-binding proteins. These results led to the hypothesis that ATP hydrolysis may serve as a biological clock in living cells. The maturation of the filaments is sensed and reflected by the change in the nucleotide state, and thus in the conformation of actin filaments [Allen et al., 1996]. As treadmilling is considered to be the driving force in actin polymerization driven biological processes it seems logical to assume that ATP hydrolysis is critical for these force generating mechanisms through its role in controlling treadmilling [Bugyi and Carlier, 2010]. Considering that the nucleotide-dependent conformational differences manifest mainly at smaller level structural changes in F-actin, it is likely that they play important roles in the establishment of the bound nucleotide-based selectivity of molecular interactions with the binding partners of actin.

## Cooperativity and Allosteric Interactions in Actin Filaments

In many cases, when the conformation of actin filaments is changed upon ligand-binding, the effects propagate along the filaments through the interaction of neighbouring actin protomers, i.e., through long-range allosteric interactions. Such allosteric interactions were reported for many actin-binding compounds [Oosawa, 1972; Miki et al., 1982a; Drewes and Faulstich, 1993; Prochniewicz et al., 1993; Muhlrad et al., 1994; Orlova et al., 1995; Selden et al., 1998; Steinmetz et al., 1998; Moraczewska, 2002] and are often called cooperativity in the special case of actin. It should be emphasized though that in this context the meaning of cooperativity deviates from the classical one, it denotes the molecular mechanism in which the ligand-induced conformational changes propagate along the filaments and appear in actin protomers distant from the location of the bound ligand. Except for a few examples—like muscle regulation [Butters et al., 1993]—the biological function attributed to the cooperativity in actin remained ambiguous. A possible function of these interactions could be coupled to the regulatory role of the conformational dynamics of actin filaments, which is controlled and finely tuned by actin-binding proteins. In many cases the proper regulation requires the conformational changes to propagate through the whole actin filaments. Due to cooperative allosteric interactions such large scale conformational changes do not require the saturation of the binding sites of the actin-binding proteins, which serves the economic functioning of the cell by allowing lower intracellular actin-binding protein concentrations to be effective for this purpose.

Several actin-binding natural products that have cytotoxic activity exhibits cooperativity [Wieland and Faulstich, 1978; Schiavo and van der Goot, 2001; Allingham et al., 2006]. Although they have obviously minor physiological importance due to their poisonous effect, the mechanisms by which they interact with actin could serve as relatively simple model systems to describe the binding of other physiologically relevant compounds. Phalloidin, a cyclic hexapeptide from the poisonous mushroom *Amanita phalloides*, is the best characterised so far. Phalloidin binds tightly to actin filaments, reduces the rate of *P*_*i*_ release [Dancker and Hess, 1990] and block the dissociation of monomers from filament ends [Dancker et al., 1975; Estes et al., 1981], which results in a more stable F-actin structure [Faulstich et al., 1977; Miyamoto et al., 1986; Isambert et al., 1995]. As the binding-site of phalloidin involves the interface of three neighbouring protomers between the two long-pitch helix of F-actin [Lorenz et al., 1993; Oda et al., 2005], it can increase the strength of interstrand protomer contacts by stapling the protomers. The cooperative nature of the binding of phalloidin to the actin was proposed when experiments showed that substoichiometric amount of phalloidin restored the elevated steady-state ATPase activity induced by the truncations in the C-terminus by limited trypsinolysis [Drewes and Faulstich, 1993]. It was further corroborated by structural [Orlova et al., 1995] and spectroscopic studies [Nyitrai et al., 2000; Visegrady et al., 2004; Visegrady et al., 2005]. Site-directed spin-labelled EPR measurements showed that phalloidin binding causes an increase in the number of interacting probes along interstrand interfaces between hydrophobic loop residues and the C-terminus of protomers, which indicates stronger contacts between the two long-pitch helix in phalloidin-decorated actin filaments [Scoville et al., 2009]. On the other hand, phalloidin does not induce local changes in the conformational dynamics of the protein matrix around hydrophobic loop, D-loop and C-terminal residues [Scoville et al., 2009]. A quantitative description of the phalloidin induced cooperative changes revealed that binding of one drug molecule changes the conformation of 7 actin protomers [Visegrady et al., 2005]. Interestingly, DSC analysis showed that the stabilising effect of phalloidin is not cooperative in the case of F-ADP-*P*_*i*_-actin (mimicked by BeF_3_-ADP or AlF_4_-ADP nucleotide analogues), which suggests different interprotomer interactions in F-actin in the ADP-*P*_*i*_ state compared to the ATP-, or ADP state [Orban et al., 2008a]. The filament stabilisation effect of phalloidin is extensively used in in vitro studies where the actin concentrations needed to be low, close or below the critical concentration for actin assembly [Kurzawa and Geeves, 1996]. Fluorescent derivatives of phalloidin are also applied to visualize the architecture of the actin cytoskeleton by fluorescence microscopy methods in intracellular studies [Small et al., 1999].

Another cyclic peptide, jasplakinolide—that can be found in a marine sponge (*Jaspis johnstoni*)—also binds to actin filaments competitively with phalloidin [Bubb et al., 1994]. Jasplakinolide accelerates actin polymerization [Bubb et al., 1994], promotes actin polymerization under nonpolymerizing conditions and lowers the critical concentration of actin assembly in vitro [Bubb et al., 2000]. Although phalloidin can stabilize actin oligomers, a similar effect of jasplakinolide was not observed [Spector et al., 1999]. Another important difference between the two drugs is that in contrast to phalloidin, jasplakinolide readily enters mammalian cells [Spector et al., 1999].

Apart from these examples many other poisonous chemicals (such as for example jararhagin) [Costa and Santos, 2004; Fenteany and Zhu, 2003] and bacterial toxins (such as clostridial binary toxins (Iota and C2 families) from *Clostridium difficile*, *Clostridium sordellii* and *Clostridium novyi*, cytotoxic necrotic factor from *Escherichia coli*, enterotoxin of *Bacteroides fragilis, Staphylococcus aureus* alpha-toxin, Shiga toxin, cytotoxic necrotizing factor type 1, *Escherichia coli* heat-stable toxin, botulinum and tetanus neurotoxins and *S. aureus* toxic-shock syndrome toxin) can alter the regulation of the cytoskeleton and/or bind directly to actin [Richard et al., 1999; Schmitt et al., 1999]. Although these have been much less characterized than phalloidin, there is a promise that as a result of future investigations the application of these compounds could lead to new medical approaches applying actin as a therapeutic target [Giganti and Friederich, 2003].

## Actin Isoforms

The tuning of the conformation and thus cellular function of actin can be achieved by the various actin isoforms which coexist in living cells. Actin is expressed as a variety of isoforms generated by gene duplications. Yeasts have 1 actin gene [Gallwitz and Seidel, 1980], while *D. discoideum* has 17 [Romans and Firtel, 1985], *D. melanogaster* and mammals have 6 genes [Fyrberg et al., 1981; Vandekerckhove and Weber, 1978]. The human genome contains four muscle (skeletal: ACTA1, smooth: ACTA2, enteric: ACTG2, cardiac: ACTC) and two cytoplasmic (ACTB and ACTG1) actin genes [Sparrow and Laing, 2008], which encode six isoforms. Actin isoforms can be found in different quantities in distinct cell types [Sheterline et al., 1995]. Four actin isoforms are muscle specific (α: skeletal, cardiac, smooth, γ2: smooth) and involved in the contractile machinery. Another two actin isoforms (β and γ1) are structurally and functionally linked to the cytoskeleton of nonmuscle cells [Chaponnier and Gabbiani, 2004]. Actin isoforms differ only in few amino-acids and display basic inherent structural and biochemical characteristics, including the ability to assemble helical filaments from monomers, ATPase activity and nucleotide dependent dynamics. However, some properties differ quantitatively [Rubenstein, 1990; Herman, 1993].

Available structural analyses of rabbit muscle, *S. cerevisiae* and *D. discoideum* actin filaments based on 3D helical reconstruction from EM images show that these actin filaments are similar in terms of the overall three-dimensional morphology, which confirms that these parameters were conserved through the evolution [Orlova et al., 1997; Steinmetz et al., 2000]. However, thorough inspection reveals important differences. Detailed analyses showed that less extensive inter-, and intrastrand contacts were formed in *S. cerevisiae* actin filaments than in muscle actin [Orlova and Egelman, 1995; Orlova et al., 1997]. The nucleotide-binding cleft is more open in *S. cerevisiae* actin protomers than in muscle actin [Orlova et al., 1997], which is consistent with the faster nucleotide exchange rate observed in yeast actin [Miller et al., 1995]. These structural differences are accompanied by altered mechanical properties. The detailed comparative analysis on the microsecond time-scale dynamics of muscle and *S. cerevisiae* actin filaments by time-dependent phosphorescence anisotropy decay measurements indicated higher torsional flexibility for yeast F-actin than for muscle F-actin [Prochniewicz and Thomas, 1999]. These results were suggested to explain that yeast actin filaments are more susceptible to fragmentation [De La Cruz and Pollard, 1996]. Hydrogen-deuterium exchange experiments reported greater exchange for *S. cerevisiae* G-actin compared to muscle actin in the barbed end, in the region of S1 and S2 and in protomer-protomer contact areas within the actin filaments as well [Stokasimov and Rubenstein, 2009]. The high degree of cooperativity and the existence of long-range allosteric interactions observed in muscle actin filaments also exist in *S. cerevisiae* F-actin [Orlova et al., 1997]. *D. discoideum* F-actin in the Ca^2+^-bound form displays less extensive interstrand contacts between the two long pitch helix than muscle actin, while these contacts are more massive in *D. discoideum* F-actin in its physiologically relevant Mg^2+^-bound form [Steinmetz et al., 2000]. The persistence length of *D. discoideum* Mg^2+^-F-actin is longer (*L*_p_ = 4.2 μm) than that of the *D. discoideum* Ca^2+^-F-actin (*L*_p_ = 1.6 μm) or the muscle Mg^2+^-F-actin (*L*_p_ = 2.3 μm). Therefore, the enhanced interstrand connectivity seems to provide the structural basis for the altered bending flexibilities of the actin filaments.

The muscle specific α-skeletal and α-cardiac actin isoforms differ only in four amino acids [Vandekerckhove and Weber, 1979; Vandekerckhove et al., 1986]. Despite of this relatively little sequence difference, the stability of the filaments differ substantially. Calorimetric (DSC) measurements revealed that muscle specific α-cardiac Mg^2+^-actin filaments were thermodynamically more stable than the α-skeletal Mg^2+^actin filaments. On the other hand, α-cardiac Mg^2+^-actin filaments are more stable than the α-cardiac Ca^2+^-actin filaments [Orban et al., 2005]. The stability of these isoforms also depend on the nucleotide state of actin. α-cardiac actin filaments polymerised from ADP-G-actin were thermodynamically less stable than the filaments of α-skeletal F-actin, while such difference was not found in filaments polymerised from ATP-actin monomers [Orban et al., 2005, 2008b]. The conformational dynamics of muscle specific actins is also sensitive to the pH. Lowering the pH resulted in a more stable protein matrix independently of the tightly bound cation. It was also suggested that the α-cardiac actin is more sensitive to the pH than the α-skeletal actin [Papp et al., 2005].

Although the experiments of recent years have shed light on many aspects of the isoform specific functional variations of actin, the understanding of how these conformational dynamics differences of polymers assembled from different actin isoforms contribute to the isoform specific functions demands further investigations.

## Actin-Binding Proteins

Apart from small ligands and peptides described above actin interacts with a large number of partner proteins. These proteins can change the conformation of actin when regulating its biological functions. Although the coupling between these structural-functional changes is not completely understood yet, there are indications that the actin-binding protein induced structural modifications are established for certain biological functions. In the next session we attempt to provide examples for these conformational changes.

### Myosins

Myosins interact with actin in all of their known biological activities. As a classic example, myosins play special and central role in the manifestation of muscle contraction [Geeves et al., 2005; Geeves and Holmes, 2005]. Muscle specific myosins are members of the myosin class II of the myosin superfamily. Other members of this class, and all the other myosin families of the large myosin superfamily (18 are known [Thompson and Langford, 2002]) express their biological effects in nonmuscle cells. It was shown that both muscle and nonmuscle myosins changed the conformation of the actin filaments upon their binding, and the myosin induced effects depended on the applied experimental conditions and also on the myosin isoforms. Time-dependent anisotropy measurements showed that in the absence of nucleotides (under rigor conditions) the binding of myosin subfragment-1 cooperatively changed the structure of actin filaments [Prochniewicz and Thomas, 1997]. The analysis of electron micrographs of actin-myosin complexes revealed that the cooperativity of the myosin binding depended on the nature of the bound cation and also on whether single or double headed myosin fragment was bound to F-actin [Orlova and Egelman, 1997]. With calcium bound to actin, the double headed muscle heavy meromyosin (HMM) showed cooperative behaviour in the absence of nucleotides, but the single headed myosin subframent-1 did not. None of the two fragments bound cooperatively to Mg^2+^-F-actin [Orlova and Egelman, 1997]. Spectroscopic methods revealed that the extent of the myosin induced conformational changes in actin depended on the nucleotide state of myosin. In the weakly bound state (when ATP or ADP-*P*_*i*_ is bound to myosin) the binding of myosin subframent-1 was not cooperative and was accompanied by a smaller change in the microsecond rotational dynamics of F-actin than in the rigor state [Prochniewicz et al., 2004]. These findings indicated that the weak-to-strong transitions—an essential step of the muscle contraction—were accompanied by conformational transitions in the actin filaments.

A recent study has found evidence that the effect of the myosin on the conformation of actin depends on the myosin isoform as well [Prochniewicz et al., 2009a]. Transient phosphorescence anisotropy decay experiments showed that both of the single headed muscle myosin subfragment-1 and nonmuscle myosin V changed the conformation of F-actin through long range allosteric interactions. However, the muscle myosin subfragment-1 decreased the rate of the intrafilament torsional motions the nonmuscle isoform had opposite effect. A detailed analysis also revealed that the length of the cooperative unit characterising the distance to which the allosteric effect of myosin binding propagated was longer for the nonmuscle myosin (6 actin protomers) than for the muscle myosin subfragment-1 (2 protomers) [Prochniewicz et al., 2009a].

The field focusing on the functions of various myosins is large and growing. It is well established that the specialised forms of myosins play essential roles in many biological functions in synergic interactions with actin. Despite the decades of investigations the information regarding the conformational effects of myosins on the structural and dynamic properties of actin filaments is limited, indicating that further investigations will be needed to properly describe and understand the biological functions of these interactions.

### Thymosin-β4

Thymosin-β4 (Tβ4) is a small (5kDa) WH2-domain (Wiskott-Aldrich syndrome protein-homology 2) containing protein. It sequesters actin monomers by forming a 1:1 nonpolymerisable complex with them, which does not participate in actin assembly at either end of the filament [Cassimeris et al., 1992]. Tβ4 preferentially binds Mg^2+^-ATP actin monomers in a nucleotide dependent manner (*K*_D_(Mg-ATP) = 2 μM, *K*_D_(Mg-ADP) = 80 μM) [(Safer et al., 1991; Carlier et al., 1993)]. At high concentrations (200–300 μM) it can be chemically cross-linked to F-actin [Carlier et al., 1996; Ballweber et al., 2002]. Tβ4 strongly inhibits the nucleotide dissociation when bound to ATP-G-actin [Goldschmidt-Clermont et al., 1992].

Extensive spectroscopic and biochemical analysis revealed that Tβ4-binding was accompanied by significant changes in the conformation of actin monomers [De La Cruz et al., 2000; Dedova et al., 2006]. Tritium exchange measurements showed that Tβ4 reduces the number of amide hydrogens that can be replaced by tritium in Mg^2+^-ATP-G-actin at intermediate rates (100–400 s), which suggests that the conformational dynamics of G-actin is restricted upon Tβ4-binding [De La Cruz et al., 2000]. Tβ4-binding substantially affects both the *Trp* and the *Tyr* band of the near-UV CD spectrum of Mg^2+^-ATP-G-actin [De La Cruz et al., 2000]. The location of several *Tyr* residues around the nucleotide binding cleft led to the proposal that the nucleotide binding cleft is narrower in the Tβ4-G-actin complex. In support of this, FRET measurements showed that Tβ4-binding decreased the distance between probes attached to *Lys*^*61*^ (S2) and *Cys*^*374*^ (S1) and to *Lys*^*61*^ (S2) and ɛ-ATP, while increased the distance between *Gln*^*41*^ (S2) and *Cys*^*374*^ (S1). This indicates that Tβ4 rotates the D-loop towards the bound nucleotide away from S1 enclosing the nucleotide binding cleft [Dedova et al., 2006]. These structural rearrangements induced by Tβ4 may reflect selective binding to the ATP-bound form of monomeric actin and its inhibitory effect on nucleotide-exchange on G-actin.

### Profilin

Profilin is an essential actin binding protein that plays important role in controlling actin dynamics and actin-based motile processes. The structure of different profilin isoforms in complex with actin isoforms were solved by X-ray crystallography [Schutt et al., 1993; Chik et al., 1996; Baek et al., 2008]. Profilin preferentially binds to the ATP-bound form of G-actin (*K*_D_ = 0.1 − 1 μM [Pantaloni and Carlier, 1993; Perelroizen et al., 1995; Eads et al., 1998; Lu and Pollard, 2001] by making contacts with the barbed face of actin monomers at the basis of S1 and S3. This structural arrangement prevents the association of profilin-actin complex with the pointed end of filaments but allows effective and exclusive assembly at the barbed end [Pollard and Cooper, 1984; Kaiser et al., 1986; Pring et al., 1992]. Profilin also catalytically accelerates the exchange of bound nucleotide on G-actin by approximately 1000-fold [Mockrin and Korn, 1980; Nishida, 1985; Goldschmidt-Clermont et al., 1992; Vinson et al., 1998].

The high-resolution crystal structure of the profilin-actin complex shows that binding of profilin to G-actin results in the rotation of the two major domains by 4.7° relative to each other in a ‘clamp’-like fashion, closing around the profilin and opening up the nucleotide-binding cleft [Schutt et al., 1993; Ferron et al., 2007; Baek et al., 2008]. The different conformational state of the nucleotide binding cleft in the profilin-actin complex was also supported by fluorescence quenching experiments that showed increased accessibility of the fluorescent ɛ-ATP in the presence of profilin [Kardos et al., 2009]. The more open protein matrix around the ATP-binding pocket in the profilin-actin complex may explain the nucleotide-exchange activity of profilin.

### ADF/Cofilin

ADF/cofilin (actin-depolymerising-factor) (AC) family of proteins can be grouped into five functionally distinct classes of actin-binding proteins (ADF/cofilin, twinfilin, Abp1/drebrin, coactosin and glia maturation factor) which are characterized by the presence of the ADF homology (ADF-H) actin-binding module [Lappalainen et al., 1998]. AC proteins are widely expressed in eukaryotic organisms and linked to the regulation of actin dynamics [de Hostos et al., 1993; Goode et al., 1998; Lappalainen et al., 1998; Wahlstrom et al., 2001; Quintero-Monzon et al., 2005; Helfer et al., 2006; Ikeda et al., 2006; Gandhi et al., 2010].

An extensively studied member of the AC protein family is ADF/cofilin that contains a single ADF-H domain. ADF/cofilin binds both G-, and F-actin, preferentially their ADP-bound form in a 1:1 and 2:1 stoichiometry, respectively [Hayden et al., 1993; Maciver and Weeds, 1994; Carlier et al., 1997; Ressad et al., 1998; Blondin et al., 2001; Galkin et al., 2001]. The activities of ADF/cofilin are regulated by pH [Yonezawa et al., 1985; Yonezawa et al., 1987], phosphorylation [Morgan et al., 1993], phosphatidylinositols [Yonezawa et al., 1985; Yonezawa et al., 1987] and other actin-binding proteins such as tropomyosin, myosin, cortactin, coronin, CAP1/Srv2p and the mammalian formin mDia1 [Nishida et al., 1984; Ono and Ono, 2002; Balcer et al., 2003; Bugyi et al., 2006; Gandhi et al., 2009; Oser et al., 2009]. ADF/cofilins from different species are qualitatively similar regarding the activities on actin, however there are differences in the magnitude of their effects [Carlier et al., 1997].

The recently solved crystal structure of twinfilin's ADF-H domain in complex with a G-actin molecule reveals that the binding interfaces are located in the groove between S1 and S3 of actin [Paavilainen et al., 2008]. The binding of ADF-H domain seems to lock the nucleotide-binding cleft between S2 and S4 in a closed conformation, which may provide the structural basis for the inhibition of the nucleotide exchange on G-actin by ADF/cofilin and also twinfilin [Nishida, 1985; Carlier et al., 1997; Andrianantoandro and Pollard, 2006; Paavilainen et al., 2008]. In agreement with structural data, quenching of the fluorescent ɛ-ATP showed decreased accessibility of the fluorophore in the presence of ADF/cofilin, which suggests that the nucleotide-binding cleft is in a more closed state in the ADF/cofilin-G-actin complex [Kardos et al., 2009]. FRET measurements revealed that ADF/cofilin-binding reduces the distance between *Gln*^*41*^ (S2) and *Cys*^*374*^ (S1) [Dedova et al., 2002] and slightly increases the distance between *Lys*^*61*^ (S2) and *Cys*^*374*^ (S1) [Blondin et al., 2001]. These results suggest that by binding to regions located in S1 ADF/cofilin induces allosteric conformational changes which cause the rearrangement of the S2 and exposure of the D-loop [Bobkov et al., 2002; Muhlrad et al., 2004].

The binding of ADF/cofilin to actin filaments exhibits a high degree of kinetic cooperativity [Hawkins et al., 1993; Hayden et al., 1993; Ressad et al., 1998; De La Cruz, 2005] and accompanied by profound and unique structural rearrangements in the actin filament. These conformational changes result in altered thermodynamic and functional properties of actin filament, including increased pointed end depolymerisation rate [Carlier et al., 1997; Ressad et al., 1998], accelerated *P*_*i*_ release from ADP-*P*_*i*_-F-actin [Blanchoin and Pollard, 1999] and inhibited binding of myosin, tropomyosin and phalloidin to ADF-decorated actin filaments [Nishida et al., 1984; Carlier et al., 1997; Ono and Ono, 2002].

The atomic model of ADF/cofilin-decorated F-actin revealed extensive contacts of the bound ADF/cofilin molecule with the outer domain of actin protomer [McGough et al., 1997; Galkin et al., 2001]. 3D helical reconstruction from EM images showed that ADF/cofilin induced a change in the filament twist by reducing the rotation per subunit by ∼5° along the short-pitch left-handed genetic helix, while maintaining a constant rise per subunit [McGough et al., 1997]. ADF/cofilin binding results in both disruption of the longitudinal contacts accompanied by a large tilt of actin subunits [Galkin et al., 2001; Bobkov et al., 2002] and the weakening of the lateral interactions in the filament [McGough and Chiu, 1999; Bobkov et al., 2004]. The shift in the filament twist was proposed to explain the ability of ADF/cofilin to promote filament disassembly [McGough et al., 1997]. However, a mutant ADF/cofilin that can change the twist of F-actin failed to enhance depolymerisation [Pope et al., 2000]. An improved analysis of EM images using a single-particle tracking based iterative helical real space reconstruction approach [Egelman, 2000] suggested that rather than imposing a new twist, ADF/cofilin freezed F-actin in an inherent, instable state, in which the contacts between the D-loop of one protomer and the C-terminal of the neighbouring protomer were disrupted [Galkin et al., 2003]. Consistently with structural studies, site-directed spin-labelled EPR detected a decrease in the number of interacting spin probes attached to *Gln*^*41*^ (S2) and *Cys*^*374*^ (S1) in neighbouring protomers and an increase in their mobility, which suggests a more flexible protein matrix around the probes and loosening of the intermonomer contacts within the long-pitch strand of F-actin [Scoville et al., 2009].

The analysis of the thermally driven fluctuations of fluorescently labelled actin filaments revealed that ADF/cofilin increased the bending flexibility and decreased the persistence length of actin filaments by 5-fold [McCullough et al., 2008]. Transient phosphorescence anisotropy measurements showed that ADF/cofilin changed the microsecond time-scale dynamics of F-actin in a long-range cooperative fashion by increasing the rate of the microsecond rotational motions and the torsional flexibility of actin filaments [Prochniewicz et al., 2005].

### Gelsolin

The gelsolin family consists of seven different proteins characterized by repeats of gelsolin-like (G) domains, including gelsolin, adseverin, villin, capG, advillin, supervillin and flightless I, which are involved in the regulation of actin dynamics [Silacci et al., 2004]. Gelsolin contains 6 tandem gelsolin-like domains and interacts with both G-, and F-actin. The interaction of gelsolin with G-actin results in the formation of a 1 : 2 gelsolin : actin complex (GA2) serving as a seed for further filament growth during which gelsolin caps the barbed end of the filament [Way et al., 1989]. Gelsolin also rapidly binds to F-actin which results in short filaments due to its slow Ca^2+^-dependent severing activity, with gelsolin remaining bound to the barbed end of the severed filament [Hesterkamp et al., 1993]. Phosphoinositides (PIP2) inhibit the severing activity of gelsolin and dissociates gelsolin from actin [Janmey et al., 1987].

Helical reconstruction of cryo-EM images showed that by binding to the side of F-actin gelsolin bridges two neighbouring actin protomers within the short pitch helix, and induces distortions within the actin filament which may sufficiently weaken the noncovalent interactions to break and sever the filaments [Bearer, 1991; McGough et al., 1998]. EM reconstructions revealed that gelsolin also induced a significant conformational change within the actin filament when it was bound to the barbed end [Orlova and Egelman, 1995]. Time-dependent phosphorescence anisotropy measurements further showed that binding of a single gelsolin molecule to the barbed end altered the conformational dynamics of the whole filament through long-range allosteric interactions resulting in increased torsional flexibility [Prochniewicz et al., 1996b]. Gelsolin binding also modifies the conformation of the C-terminus in the vicinity of *Cys*^*374*^. Further cross-linking and fluorescence measurements showed that the nucleation by gelsolin was promoted by conformational changes between the D-loop and the C-terminal of protomers, which propagate along the filament from the gelsolin capped barbed ends [Khaitlina and Hinssen, 1997].

### Actin Nucleation Factors

Both in vitro and in vivo, the rate of the spontaneous polymerisation of actin is limited by the instability of the initial actin dimers/trimers. In cells, to overcome this kinetic barrier and regulate precisely the spatiotemporal initiation of actin structures, membrane-associated stimuli-dependent nucleation factors catalyze the de novo formation of actin filaments by unique mechanisms. The first identified nucleation machinery includes the WASP/WAVE/Scar proteins (Wiskott-Aldrich syndrome protein/WASP family verprolin homologous/suppressor of c-AMP response) which activate the Arp2/3 complex to generate a branched daughter filament from a pre-existing mother filament [Pollard, 2007]. Recently, the repertoire of actin nucleation factors has been extended to proteins containing multiple WH2-domains, like Spire [Quinlan et al., 2005], Cordon-bleu [Ahuja et al., 2007], Leiomodin [Chereau et al., 2008] and VopF/VopL [Liverman et al., 2007]. These proteins are thought to bind actin monomers via the WH2-repeats and stabilise their complexes. A third class of nucleation factors is the formin proteins which use the formin homology domains (FH1 and FH2) to nucleate actin assembly and drive processive barbed end growth of profilin-actin by associating persistently with the elongating barbed end and simultaneously enabling subunit addition [Goode and Eck, 2007].

The mechanisms by which nucleation factors catalyze filament assembly and regulate barbed end dynamics has been extensively studied over the past few years. For detailed information we direct the readers to recent reviews [Kerkhoff, 2006; Goode and Eck, 2007; Pollard, 2007; Renault et al., 2008; Bugyi and Carlier, 2010]. In contrast, very little is known about the conformational dynamics of the filaments nucleated by these factors. Formins were recently the first to be shown to alter the conformational dynamics of actin filaments. Temperature-dependent FRET and time-dependent fluorescence anisotropy measurements revealed that the mammalian formin Dia1 (mDia1) by binding to barbed ends increased the overall flexibility of actin filaments, which results from the more tenuous interactions between neighbouring protomers in formin-nucleated actin filaments [Bugyi et al., 2006; Papp et al., 2006]. The FH2 domain is sufficient for this conformational change [Ujfalusi et al., 2009]. A detailed analysis revealed that the formin-induced conformational changes propagated through several hundreds of nanometres from the barbed end via long-range allosteric interactions [Bugyi et al., 2006; Papp et al., 2006]. An extended description by ST-EPR measurements showed decreased torsional flexibility of formin-nucleated actin filaments [Kupi et al., 2009], suggesting that the formin-induced changes in the conformational dynamics of actin filaments are complex. More importantly, the formin-nucleated, more flexible actin filaments altered functional properties. They decreased the thermal stability, increased the phosphate release rate and altered the interactions with ADF/cofilin [Bugyi et al., 2006]. Time-dependent anisotropy measurements showed that tropomyosin or myosin restore the formin-induced conformational changes and stabilise the formin-nucleated actin filaments [Ujfalusi et al., 2009] and unpublished observations). Bni1 but not Bnr1 from *S. cerevisiae* was also proposed to induce conformational changes within yeast actin filaments, which was indicated by the increase of the pyrene excimer fluorescence in formin-nucleated actin filaments [Wen and Rubenstein, 2009].

## Mutations in Actin and Pathologies

Minor changes in the highly conservative amino-acid composition of actin can have deep impact on the structure of actin, its function and interactions with its partner molecules (e.g., myosin, tropomyosin) [Bookwalter and Trybus, 2006]. Mutations in the actin encoding genes, which are dominant missense mutations in most of the cases, lead to severe dysfunction of the related actin structures and diseases. The highly controlled site directed mutagenesis of actin in the recombinant baculovirus/Sf9 system give a crucial tool to acquire detailed information about the structural/functional changes caused by mutations [Joel et al., 2004; Rould et al., 2006; Debold et al., 2009] and to understand the molecular mechanism behind the actin-related pathological situations.

Mutations in actin are frequently manifested in the form of skeletal [Sparrow et al., 2003; Feng et al., 2009] and cardiac myopathies [Olson et al., 1998, 2000; Mogensen et al., 2004]. The mutant skeletal α-actins can sometimes accumulate into compact intranuclear structures [Ilkovski et al., 2005]. In the case of actin mutations resulting in myopathy and mostly of intranuclear rod myopathy, the mutated amino acids are clustered around the central cleft of actin. Amino acid mutations related to nemaline myopathy were dispersed around the actin molecule affecting a large number of different binding sites (e.g., actin-actin, acto-myosin, actin-tropomyosin, actin-nebulin, actin-α-actinin) [Sparrow et al., 2003]. Single amino acid mutations can cause both structural and functional defects. Mutations of L94P, E259V result in impaired folding while the I357L mutant exhibits a less compact protein conformation [Ilkovski et al., 2004]. The I64N, Q263L, G268C, G268R and N280K mutants have a lower capacity to copolymerize with wild type actin [Sparrow and Laing, 2008; Sparrow et al., 2003]. The R183G nemaline mutant actin has reduced polymerization capabilities [Ilkovski et al., 2004].

Under in vitro conditions, the M132V nemaline actin mutant obtained from human biopsies was demonstrated to have lower polymerization capability and increased velocity in the actomyosin motility assay [Marston et al., 2004]. Mutations in the ACTG1 gene coding cytoskeletal actin components were related to a human disease (dominant progressive deafness) at first in 2003 [Zhu et al., 2003]. The substitution of the amino acids (T89I, K118M, P332A, P264L) disturbed the primary (mutation P332A) and secondary myosin binding sites (mutation T89I), the binding sites for α-actinin (mutation K118M) and a region that has a role in the stability or compliance of the actin filaments (mutation P332A) [Zhu et al., 2003]. The authors proposed that these mutations could modify the γ-actin related functions of the affected cells causing the progressive hearing loss of the patients.

The T278I mutation of the ACTG1 gene coded cytoskeletal γ-actin can also cause autosomal dominant hearing loss [van Wijk et al., 2003]. In this case the authors proposed that the mutation of the cytoskeletal actin components produced structural changes and impaired polymerisation properties as well which affected the normal function of the hair cells in the inner ear [van Wijk et al., 2003]. The effect of the γ-actin mutations, which were identified in the autosomal dominant nonsyndromic hearing loss on the fuction of actin was studied biochemically as well with the conclusion that the impaired regulation of the actin filaments by the actin-binding proteins could be the key factor in the development of the deafness [Bryan et al., 2006]. It was suggested that cofilin as an important regulator of actin filament turnover could be involved to balance the instability of the filaments caused by the point mutations [Bryan and Rubenstein, 2009]. It was also concluded that the progressive disintegration of the cytoskeleton within the hair cells would result in the loss of hearing among the patients suffering from this disease [Bryan et al., 2006; Bryan and Rubenstein, 2009; Morin et al., 2009].

**Fig. 4:**
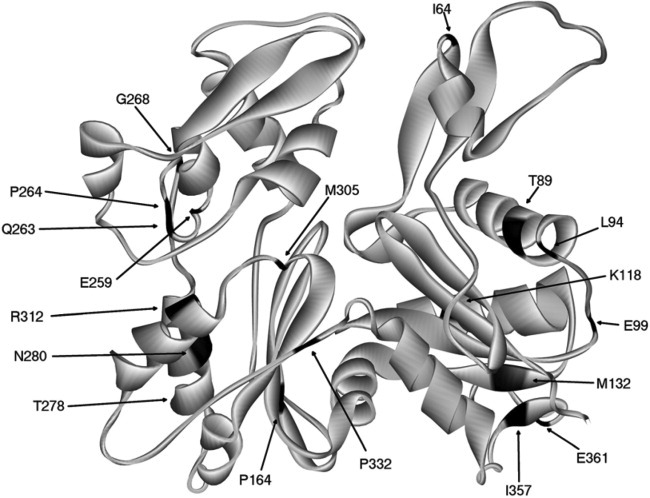
The most common mutation sites in muscle actin The most common mutation sites are labeled with one letter and ID number of the amino acids [Olson et al., 1998; van Wijk et al., 2003; Zhu et al., 2003; Ilkovski et al., 2004; Marston et al., 2004]. The ribbon structure of rabbit skeletal muscle actin (PDB code: 1ATN [Kabsch et al., 1990] is colored black at the mutated points.

In the case of the familial hypertrophic cardiomyopathy some mutant actin (Q99K, P164K, M305L) presented slower folding in vitro. The incorporation of the monomers into the filamental structure affected adversely as well [Vang et al., 2005]. The familial dilated cardiomyopathy is etiologically linked to R312H and Q361G mutations, which impair the interaction between the cardiac α-actin and the intercalated disc, Z-disk, α-actinin and dystrophin [Kuhlman et al., 1992; Levine et al., 1992; Olson et al., 1998]. The most common mutation sites are shown in Figure 4.

Besides mutations in actin itself, mutations in genes encoding actin-associated proteins also lead to genetic conditions. Dystrophin and its homologous utrophin are members of the spectrin-superfamily. These proteins are associated with the costameric cytoskeleton in striated muscle that circumferentially locates around the myofibrils in register with the Z-disk and physically couples the force-generating myofibrils to the sarcolemma [Ervasti, 2007]. The mutation of the dystrophin gene causes Becker and Duchenne muscular dystrophy and dilated cardiomyopathy characterised by the rapid progression of muscle degeneration and associated defects in the elasticity and integrity of the sarcolemma [Petrof et al., 1993; Puttini et al., 2009]. Both dystrophin and utrophin contains amino terminal tandem calponin homology (CH) domains and spectrin-like repeats which provide extended contact with the actin filament [Blake et al., 2002; Ervasti, 2007]. In spite of these similarities, structural reconstructions and biochemical studies suggest that dystrophin and utrophin have different mode of association to F-actin [Sutherland-Smith et al., 2003; Rybakova et al., 2006].

The effects of dystrophin and utrophin on the structural dynamics of actin filaments were studied with transient phosphorescence anisotropy. The results showed that both of these proteins altered the rotational dynamics of actin filaments [Prochniewicz et al., 2009b]. However the effects of utrophin are more pronounced than that of dystrophin, even though dystrophin makes more extensive contacts with F-actin [Sutherland-Smith et al., 2003]. Binding of utrophin to F-actin resulted in the decreased amplitude of both intraprotomer, torsional and bending motions in actin filaments while increased the rate of these motions, which indicates the increased torsional flexibility of actin filaments. This unusual combination of effects on the rotational dynamics indicates that binding of utrophin and dystrophin attribute special mechanical properties to F-actin; making them stronger and more flexible. The changes in actin filament flexibility induced by these actin-binding proteins may have importance in optimizing the mechanical properties of the costamers, allowing to laterally transmitting forces from the sarcomere to the extracellular matrix during muscle contraction or stretch, while damping the stress imposed on the sarcolemma.

## Biological Relevance: An Example

It is difficult to pinpoint in a simple model the biological function related to the conformational changes in actin monomers or filaments. The difficulty comes from two sources. Actin has various and complex biological functions and apparently it can effectively and relatively quickly adapt to many intracellular situations and binding partners. On the other hand there are a large number of molecules from small cations to proteins which contribute to the broad conformational landscape of actin. While these conformational changes are extensively studied in vitro, very little is known about the conformational dynamics of actin in the cellular environment. One of the aims of the previous sections of this review was to provide examples of these specific interactions and environments, and to give an overview regarding the role of the conformational state of actin.

Although the described interactions were specific some of them could provide bases for a more general model regarding the regulatory function of actin conformation. Recent observations suggested that formins, a group of actin nucleation factors, could substantially change the conformation of actin filaments by making them more flexible [Bugyi et al., 2006; Papp et al., 2006]. The changes were reversed by the binding of other actin-binding proteins such as tropomyosin [Ujfalusi et al., 2009] or myosin (our unpublished observations). It is well established that certain groups of actin-binding proteins preferentially localise to actin structures generated by the Arp2/3 complex, while other actin-binding proteins bind formin nucleated actin filaments [Moseley and Goode, 2006; Sirotkin et al., 2005]. However, the mechanism by which the actin-binding proteins are distinguished and selected by the filaments is unknown, so is the function of this interesting selection process in living cells. What seems to be obvious though is that the actin nucleation factors modify the affinities of the actin binding proteins for the actin filaments to achieve the molecular selection. One of the most plausible ways to tune the affinities would be to change the conformation of the actin filaments. The existence of this molecular mechanism seems more reasonable if one considers the timing of the protein–protein interactions. Due to their nature actin nucleation factors are the first proteins to establish contact with the forming actin filaments. The observations that formins can alter the structural state of the actin filaments support this idea. Although further clarifications and supporting experiments are required at the current state of our understanding it appears that the actin nucleation factor induced modifications in the actin filaments could play central role in the regulation of the formation of actin associated intracellular protein complexes.

## Future Perspectives

So far, the conformational dynamics of actin, and the effects of different factors were investigated in depth under in vitro conditions. Considering the rich variety of actin functions and the large amount of data accumulated in these studies some of the couplings between the structural changes and biological functions were revealed. In many other cases the complete understanding of the roles of intramolecular mechanisms in actin demands further studies. Cellular actin networks interact simultaneously with more proteins that can induce different changes in the conformational dynamics of individual actin monomers or filaments. How are these changes superimposed—enhanced or dampened—and determine the overall conformational dynamics of actin networks? How does the conformational dynamics of actin filaments play a role in the establishment of the functional properties of relevant actin networks? Recent advances in the development of novel technologies (such as fluorescence lifetime imaging microscopy (FLIM) or fluorescence anisotropy decay imaging microscopy (FADIM) [Suhling et al., 2005] open the possibility to study the conformational dynamics of actin in specific cellular structures associated to diverse regulatory proteins. These in vivo measurements will be essential to understand how actin conformational dynamics is regulated and contributes to the functional and dynamic segregation of actin networks.

## Glossary

### Rotational Correlation Time (φ)

describes the rotational diffusion of a molecule, it characterises the time-dependence of the orientation-dependent spectroscopic observable. The observable depends on the technique used to study the conformational dynamics of the molecule: in fluorescence/phosporescence spectroscopy it is the transition moment of the probe, in EPR it is the orientation of the spin label. The rotational correlation time inversely related to the rate/diffusion coefficient (θ) of the rotation. The rotational correlation times describing the rotational motion of G-, or F-actin are distributed to a broad time scale; from *fs* to *ms* and can be measured by different approaches (see Fig. 2).

### Torsional Rigidity of F-Actin

mechanical property of actin filaments, it measures the resistance of the filament to an external twisting torque.

### Flexural (Bending) Rigidity of F-Actin

mechanical property of actin filaments, it measures the resistance of the filament to bending forces.

### Persistence Length (*L*_p_)

describes the flexural rigidity of actin filaments. It equals the arc length of the filament over which the tangent angle at every point along the arc length correlates in three-dimensional motion. The persistence length is the distance over which the filament bends due to thermal fluctuations. The persistence length is related to the flexural rigidity (*K*) of F-actin by the following equation:


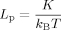


where *k*_B_ is the Boltzmann-constant and *T* is the absolute temperature. Actin filaments belong to the semiflexible polymers with typical persistence length of 0.1 – 20 μm.
